# Assessing the Satisfaction of Physicians in Using the Electronic Prescription System of Social Security in Iran: A Cross‐Sectional Study

**DOI:** 10.1002/hsr2.71473

**Published:** 2025-11-26

**Authors:** Mohammad Reza Mazaheri Habibi, Narges Norouzkhani, Fatemeh Moghbeli, Mahdieh Mohammadi Majd, Gholamreza Moradi, Masood Setoodefar

**Affiliations:** ^1^ Department of Health Information Technology Varastegan Institute for Medical Sciences Mashhad Iran; ^2^ Department of Health Information Technology, School of Management and Medical Informatics Tabriz University of Medical Sciences Tabriz East Azerbaijan Iran; ^3^ Department of Computer Sciences, Faculty of Engineering Khayyam University Mashhad Iran

**Keywords:** cross‐sectional study, electronic Prescription system, Iran, physician satisfaction, social security

## Abstract

**Background and Aims:**

Electronic prescribing (e‐prescribing) has emerged as a vital strategy to enhance medication safety, reduce healthcare errors, and streamline clinical workflows. However, its success is largely dependent on user acceptance and satisfaction. This descriptive, exploratory cross‐sectional study evaluated physicians' satisfaction with the Social Security Organization's electronic prescription system in two major cities of northeastern Iran—Mashhad and Sabzevar—where the system has been widely implemented in outpatient settings.

**Methods:**

A descriptive cross‐sectional study was conducted in 2021 involving 60 physicians selected via convenience sampling. Data were collected through a 36‐item researcher‐designed questionnaire, which assessed satisfaction with system performance, training quality, technical support, and expected benefits. The questionnaire demonstrated strong reliability (Cronbach's alpha = 0.918) and was validated by a panel of health IT experts and physicians. Data were analyzed using SPSS version 21. Group comparisons and correlation analyses were performed based on experience level, system usage duration, and self‐reported computer proficiency.

**Results:**

Respondents were predominantly 30–39 years old and reported high levels of digital literacy and motivation to adopt new technologies. Satisfaction was particularly high in areas such as prescription error reduction and the ability to transmit prescriptions to pharmacies electronically. However, a significant proportion of physicians expressed dissatisfaction with system speed, particularly with login and data entry times, and with the lack of adequate training. This led to a notable contradiction where 46.7% of participants acknowledged the system's effectiveness in reducing errors, yet the same proportion indicated they would not recommend it to colleagues. Expectations for improved patient care, workflow integration, and access to medication histories were also emphasized.

**Conclusion:**

Although the system demonstrated key functional benefits, significant limitations in technical performance, interface usability, and user support infrastructure hindered full acceptance. These findings offer initial, context‐specific insights and may serve as a foundation for broader, multi‐regional evaluations of e‐prescribing systems in Iran. Addressing these shortcomings through targeted training, interface optimization, and performance enhancements is critical to advancing physician satisfaction and long‐term system integration.

**Abbreviations**
e‐prescribing: electronic prescribing
IT: information technology
SPSS: Statistical Package for the Social Sciences
TAM: Technology Acceptance Model

## 1 Background

Over the past decades, handwritten prescriptions have been a standard communication method between physicians and other healthcare providers in diagnostic and therapeutic workflows [1]. This traditional process, performed manually using pen and paper, is prone to human error, illegible handwriting, and incomplete documentation, which can significantly compromise patient safety [2, 3]. Numerous studies have demonstrated that medication errors—such as incorrect dosage, drug selection, or misinterpretation—are directly linked to prescription‐related issues and contribute to preventable adverse events and mortality [4, 5]. In the United States alone, medical errors are estimated to result in approximately 180,000 deaths annually [6].

Such errors not only endanger patient safety but also increase healthcare costs, extend hospital stays, and, in some cases, lead to permanent disability or death [7, 8, 9]. In response to these risks, electronic prescribing (e‐prescribing) systems have emerged as a technology‐driven alternative to reduce prescription‐related errors, enhance clarity, and streamline workflows [10, 11].

E‐prescribing systems have been shown to improve the quality of healthcare services by minimizing human errors, supporting clinical decision‐making, and facilitating the efficient transmission of accurate prescriptions [12, 13, 14]. These systems also enhance interoperability between providers and pharmacies by delivering structured and legible prescriptions in real time [15]. Clinical decision support features—such as alerts for drug‐drug interactions, contraindications, and dosage warnings—have further strengthened their role in improving prescribing accuracy and safety [16, 17, 18].

Moreover, e‐prescribing addresses challenges such as duplicate prescriptions, illegible handwriting, and time delays associated with paper‐based workflows. These enhancements benefit physicians, pharmacists, and patients by reducing administrative burdens and improving medication access [19, 20, 21].

Despite these advancements, the effectiveness of any e‐health system largely depends on end‐user satisfaction. Physicians' acceptance, perceived ease of use, and trust in system reliability are critical for successful and sustainable implementation [16, 22, 23, 24, 25]. Therefore, assessing user satisfaction is not merely a quality assurance step but a strategic component of system optimization. Prior studies have emphasized the importance of user feedback in improving system design and reducing resistance to adoption [1, 26].

The Social Security Organization launched a nationwide electronic prescription system in Iran in late 2016. Initially introduced in a hospital setting, it has since expanded to clinics and outpatient facilities nationwide. While the system has been operational for several years, there remains a critical lack of research evaluating physicians' satisfaction with its functionality, usability, and overall impact on clinical workflows. Most existing research in Iran has focused on technical infrastructure or general attitudes toward health IT, with limited empirical data on physicians' lived experiences and performance evaluations [27].

Internationally, countries such as Saudi Arabia, Australia, and Germany have systematically evaluated physician satisfaction with e‐prescription systems and used those insights to enhance system design [28, 29, 30]. However, comparable localized research in Iran is scarce. This study addresses that gap by comprehensively assessing physician satisfaction with the Social Security Organization's electronic prescription system in Mashhad and Sabzevar—two cities where system usage is well established.

The significance of this study lies in its potential to reveal usability strengths, highlight critical pain points, and offer targeted recommendations to improve the system's effectiveness and user engagement. Given the direct link between prescription errors and patient outcomes, improving system satisfaction could lead to measurable enhancements in clinical safety and efficiency. This is the first study to evaluate physician satisfaction with Iran's national Social Security e‐prescribing system, offering actionable insights for health IT developers, policymakers, and administrators.

## 2 Methods

### 2.1 Study Design

This study was conducted in 2021 as a descriptive, cross‐sectional, and applied survey to evaluate physicians' satisfaction with the electronic prescription system implemented by the Iranian Social Security Organization. Sixty physicians who were actively using this system in outpatient clinics in Mashhad and Sabzevar were invited to participate. Owing to practical constraints and the limited pool of eligible users, convenience sampling was used to recruit participants. The survey examined physicians' perceptions of system performance, training and support quality, user‐interface usability, and expectations for future improvements.

### 2.2 Study Population and Sampling

The study included 60 physicians from Mashhad and Sabzevar actively using the Social Security Organization's electronic prescribing system in outpatient clinics. A convenience sampling approach was employed due to practical constraints, including time limitations, accessibility, and the need to recruit active users of the system. While convenience sampling may introduce selection bias, it is a common approach in exploratory cross‐sectional surveys where rapid recruitment is needed from a defined user base [27]. This method enabled the inclusion of a range of participants from multiple clinics, aiming to capture variation in clinical experience, system usage, and technological proficiency.

#### 2.2.1 Inclusion and Exclusion Criteria

Eligible participants were physicians with at least 6 months of continuous experience using the electronic prescription system and were actively engaged in outpatient care during the data collection period. Physicians with less than 6 months of system exposure, those with irregular usage patterns, and those who declined to participate or withheld consent were excluded from the study. Participants were selected to ensure variation in professional experience, self‐assessed computer skills, and duration of interaction with the system.

#### 2.2.2 Sample Size

A total of 60 physicians participated in the survey. The sample size was determined by the number of eligible physicians available during the 2‐month data collection period, rather than through a formal power calculation, as the study's aim was descriptive and exploratory rather than inferential. Given the exploratory nature of this study and the limited number of physicians actively using the system in the target cities (Mashhad and Sabzevar), a sample of 60 participants was deemed sufficient to generate preliminary insights. This approach is consistent with descriptive health IT research conducted in defined contexts, where the primary objective is to capture usability patterns and initial experiences rather than to achieve national representativeness. Comparable satisfaction surveys on electronic prescribing systems have used relatively small sample sizes and convenience sampling, given the defined user base e.g. [16, 27, 31]. Thus, a sample of 60 was considered sufficient to provide initial insights and identify areas for improvement, although we acknowledge that it limits statistical power and generalizability [24].

### 2.3 Setting

Participants were recruited from clinics and outpatient centers affiliated with the Iranian Social Security Organization in *Mashhad* and *Sabzevar*. These locations were selected because they represented early adopters of the electronic prescribing system and provided an appropriate mix of clinical disciplines and patient volumes to support comprehensive feedback.

### 2.4 Questionnaire Design

A customized researcher‐developed questionnaire was employed due to the absence of a validated instrument tailored to the context of Iranian electronic prescribing. The questionnaire was designed through a structured process involving a review of prior studies evaluating user satisfaction with health IT systems e.g. [16, 27, 31]. and expert consultation with five health IT specialists and four experienced physicians to establish face validity. Experts were selected based on their academic background, professional experience with electronic health systems, and familiarity with questionnaire development. Feedback from this panel led to iterative revisions that improved clarity, cultural relevance, and content alignment with the study objectives. While face validity was confirmed, we acknowledge that a limitation of this study is that more comprehensive psychometric testing (e.g., content validity index, construct validity analysis) was not performed. Internal consistency reliability was assessed using Cronbach's alpha, yielding a value of 0.918, indicating excellent internal reliability.

Data analysis and reliability testing were performed using SPSS version 21. Total Satisfaction was defined as the combined percentage of “Satisfied” and “Very Satisfied” responses; similarly, Total Dissatisfaction combined “Dissatisfied” and “Very Dissatisfied.”

### 2.5 Data Collection Procedure

Data collection occurred over 2 months. Questionnaires were distributed in person by trained staff at participating health centers. All physicians received and signed informed consent forms before participation. They were given adequate time to complete the questionnaire privately to reduce response bias. Completed responses were submitted anonymously. Confidentiality was assured throughout the process, and data collection protocols were standardized to minimize interviewer bias and external influence.

### 2.6 Subgroup Analysis

To explore how various factors influenced satisfaction levels, physicians were categorized based on years of clinical experience, duration of system usage, and self‐assessed computer literacy. These subgroupings enabled comparative analyses to determine whether professional background or technological proficiency affected users' perceptions of the system.

### 2.7 Data Analysis

Quantitative data were analyzed using IBM SPSS Statistics, version 21. Descriptive statistics summarized demographic characteristics and satisfaction scores, including frequencies, means, percentages, and standard deviations. Pearson's correlation coefficients were calculated to evaluate relationships between computer skills and system satisfaction variables. Group comparisons were conducted using independent‐sample *t*‐tests and one‐way ANOVA, depending on the number of categories involved. Normality assumptions were tested before parametric analyses, and a significance threshold of *p* < 0.05 was applied to all tests.

Open‐ended responses were analyzed using thematic analysis based on Braun and Clarke's framework. Two independent reviewers generated initial codes, and themes were derived through iterative refinement and discussion. This mixed‐methods approach enriched the interpretation of satisfaction drivers and barriers.

### 2.8 Ethical Considerations

The study protocol was reviewed and approved by the appropriate institutional review board. Written informed consent was obtained from all participants. Participants were assured of the confidentiality of their responses, their right to withdraw at any time, and that no personal identifiers would be collected.

## 3 Results

### 3.1 Participant Demographics

**Figure 1:**
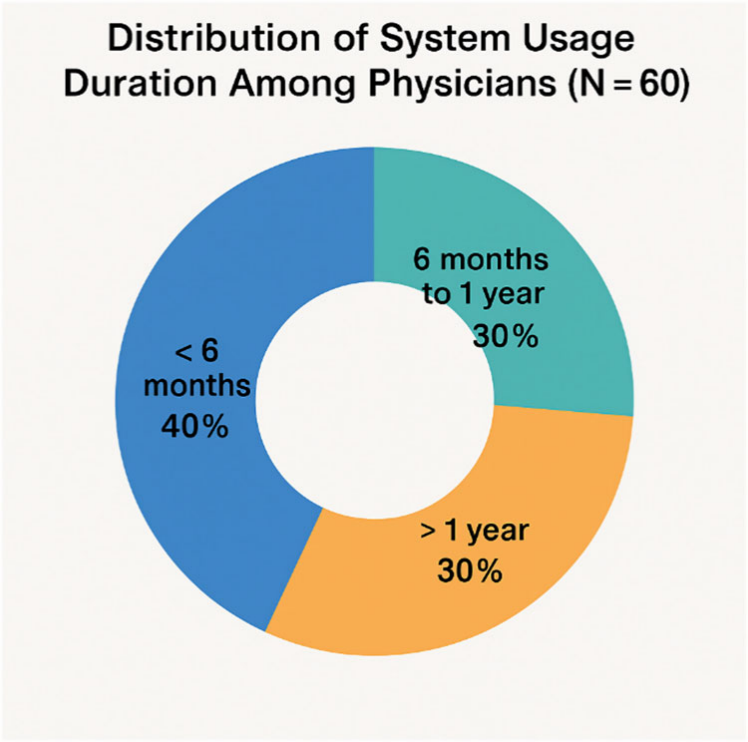
Distribution of system usage duration among physicians (N = 60).

The study sample included 60 physicians, with a relatively balanced gender distribution: 31 (51.7%) were female, and 29 (48.3%) were male. The largest age group was 40 years and above (63.9%), followed by those aged 30–39 (36.1%). Regarding professional experience, 31.7% of physicians had less than 5 years of practice, 46.7% had between 5 and 10 years, and 21.7% had more than 10 years of experience. In terms of education, 48.3% held a Doctorate in General Medicine. The distribution of system usage duration among participants is illustrated in Figure 1, indicating that 40.0% had used the electronic prescribing system for less than 6 months.

The remaining physicians were evenly divided between 6 months to 1 year and more than 1 year of experience with the system, as detailed in Table 1.

**Table 1:** Demographic characteristics of participants (N = 60).

Variable	Category	Frequency (*n*)	Percentage (%)
Gender	Female	31	51.7
	Male	29	48.3
Age group	30–39 years	21	36.1
	40+ years	39	63.9
Work experience	Less than 5 years	19	31.7
	5 to 10 years	28	46.7
	More than 10 years	13	21.7
Level of education	Doctorate in General Medicine	29	48.3
System usage duration	Less than 6 months	24	40.0
	6 months to 1 year	18	30.0
	More than 1 year	18	30.0

### 3.2 Motivation and Computer Proficiency

As presented in Table 2, 67% of physicians rated their computer skills as good or very good, while 66.7% reported high confidence in using the electronic prescribing system. Additionally, 66.6% expressed strong motivation to acquire new skills relevant to system use.

**Table 2:** Self‐assessed computer skills, ability to use the system, and motivation to acquire new skills.

Question	Very good (%)	Good (%)	Average (%)	Poor (%)	Very poor (%)
How do you rate your overall computer skills?	25.3	41.7	31.7	1.7	0.0
How do you evaluate your ability to use the electronic prescription system?	21.7	45.0	31.7	1.7	0.0
How would you assess your motivation for acquiring new skills to use a system?	18.3	48.3	23.3	3.3	6.7

**Figure 2:**
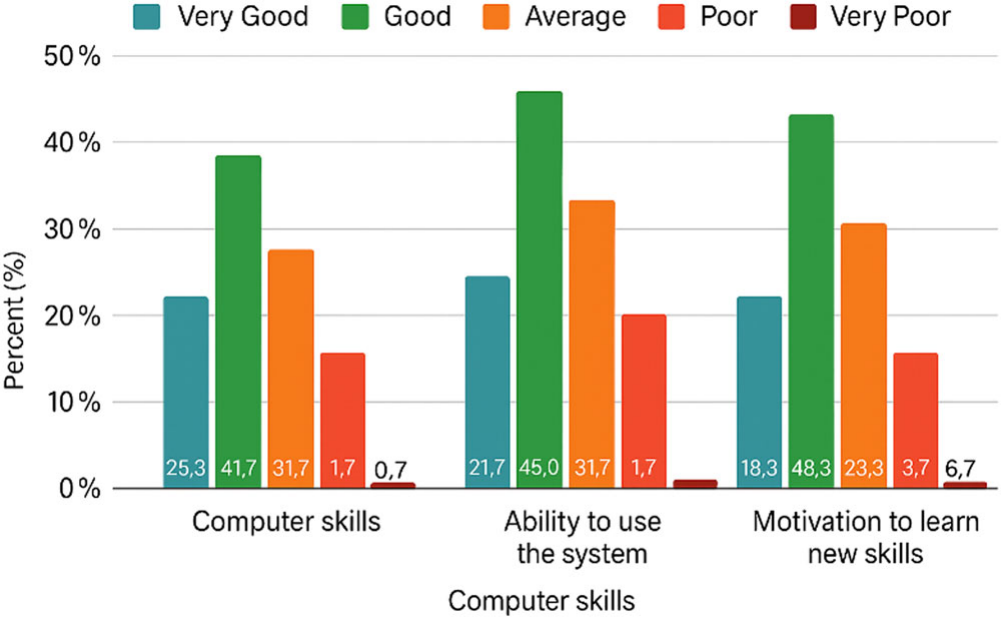
Comparison of self‐assessed skills and motivation levels among physicians (N = 60).

Figure 2 visually compares these distributions, highlighting the predominance of positive responses across all three domains. These findings suggest that digital literacy and learning motivation may have a significant impact on system engagement.

Moreover, as shown in Table 3, a significant association was observed between physicians' computer proficiency, their confidence in using the system (*r* = 0.42, *p* = 0.0004), and their motivation to acquire new skills (*r* = 0.31, *p* = 0.018). These correlations suggest that physicians with higher self‐assessed computer competency are likelier to engage effectively with electronic systems and pursue further digital skill development.

**Table 3:** Association between physicians' computer skills and their ability to use the system and motivation to acquire new skills (N = 60).

Independent variable	Dependent variable	Test used	Test statistic	*p*‐value
Computer skills	Ability to use the system	Pearson correlation	*r* = 0.42	0.0004
Computer skills	Motivation to acquire new skills	Pearson correlation	*r* = 0.31	0.018

### 3.3 Satisfaction With System Performance and Speed

Physicians expressed moderate satisfaction with core system functionalities. Specifically, 46.7% were satisfied with the system's ability to reduce prescription errors, and similar proportions appreciated the drug dosage alerts (46.7%) and drug interaction warnings (50%). However, 48.3% were dissatisfied with the system's overall performance, especially concerning responsiveness and reliability.

As shown in Table 4, satisfaction with prescription transmission to pharmacies (73.4%) and patient confidentiality (73.4%) ranked highest among functional features. In contrast, time‐related performance metrics showed the lowest satisfaction scores. Only 28.3% of physicians were satisfied with system login speed, with 53.3% reporting dissatisfaction. Similarly, only 28.3% were satisfied with the time required to enter prescriptions and patient data, while a significant 61.7% expressed dissatisfaction with this task.

**Table 4:** Physicians' satisfaction with system performance and functional features (N = 60).

Performance area	Very dissatisfied (%)	Dissatisfied (%)	Neutral (%)	Satisfied (%)	Very satisfied (%)	Total satisfaction (%)	Total dissatisfaction (%)
Alerts for incorrect dosage	8.3	16.7	28.3	31.7	15.0	**46.7**	**25.0**
Alerts for drug interactions	6.7	26.7	16.7	41.7	8.3	**50.0**	**33.4**
Impact on patient treatment quality	16.7	26.7	20.0	23.3	13.3	**36.6**	**43.4**
Overall performance and functionality	25.0	23.3	23.3	21.7	6.7	**28.4**	**48.3**
Prescription transmission to pharmacies	5.0	8.3	13.3	51.7	21.7	**73.4**	**13.3**
System login speed	30.0	23.3	18.3	23.3	5.0	**28.3**	**53.3**
Time to enter prescriptions/patient data	40.0	21.7	10.0	20.0	8.3	**28.3**	**61.7**
Speed in generating a new prescription	23.3	28.3	20.0	20.0	8.3	**28.3**	**51.6**
Speed in navigating system pages	21.7	26.7	23.3	21.7	6.7	**28.4**	**48.4**
System response to user commands	16.7	31.7	25.0	20.0	6.7	**26.7**	**48.4**
Overall ease of system use	18.3	18.3	26.7	30.0	6.7	**36.7**	**36.6**
Interface attractiveness and usability	21.7	21.7	23.3	23.3	10.0	**33.3**	**43.4**
Confidentiality of patient information	3.3	3.3	20.0	56.7	16.7	**73.4**	**6.6**

*Note:* Percentages may not total exactly 100% due to rounding. *“Total Satisfaction”* represents the combined percentage of responses marked as “Satisfied” and “Very Satisfied,” while *“Total Dissatisfaction”* reflects the sum of “Dissatisfied” and “Very Dissatisfied” responses. All totals were automatically calculated from the original response frequencies.

Notably, this performance gap is reflected in overall perceptions: although 46.7% confirmed that the system reduced prescription errors and unnecessary interventions, the same proportion stated they would not recommend the system to colleagues. This contradiction suggests that, while certain technical functions perform effectively, broader usability and support issues—particularly slow system response, login delays, and insufficient training—undermine overall satisfaction. Similar patterns have been reported in other resource‐limited healthcare settings [24].

To further explore this discrepancy, we conducted a subgroup analysis to assess whether the unwillingness to recommend the system—despite satisfaction with specific features such as error reduction—was associated with physicians' computer proficiency or duration of system use. However, no statistically significant associations were found (*p* > 0.05 for all comparisons).

One‐way ANOVA and *t*‐tests examined group differences in satisfaction scores based on physicians' characteristics. A statistically significant difference was observed for system usage duration, with *a p‐value* of 0.047. No significant difference was found for work experience (*p* = 0.180) or computer proficiency (*p* = 0.340).

### 3.4 Satisfaction With Training and Support

The analysis revealed a significant gap in satisfaction with training quality. Although 43.3% of participants reported receiving adequate training before system deployment, 55% were dissatisfied with the training related to handling system failures. This reflects a notable weakness in preparedness and technical support infrastructure.

### 3.5 Expectations for Future System Improvements

**Figure 3:**
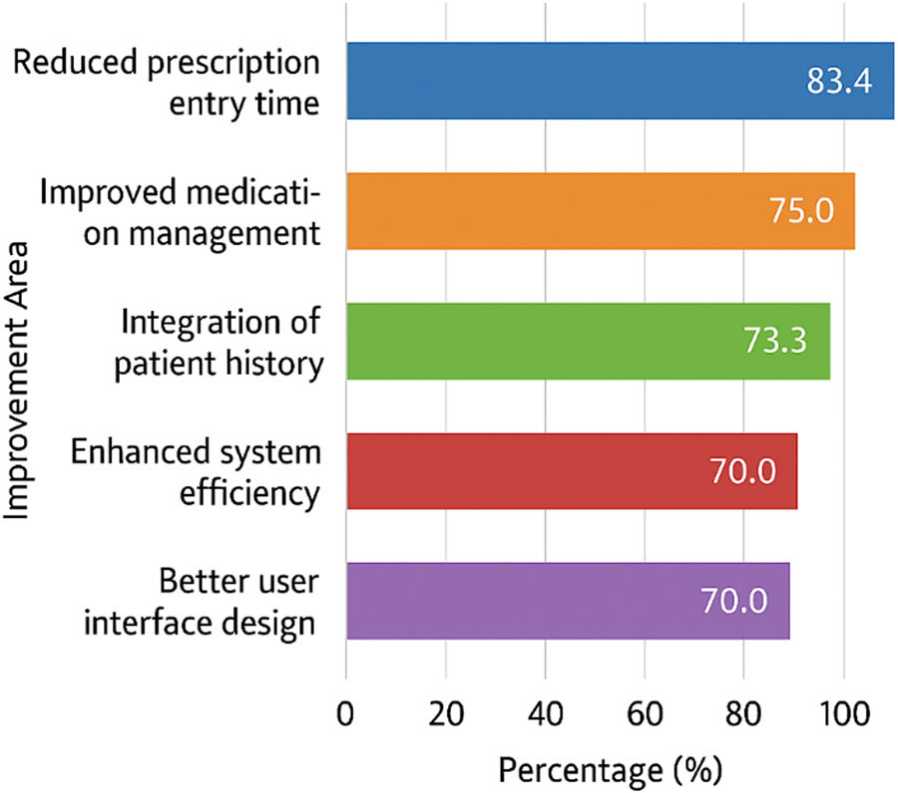
Percentage of physicians expecting improvements in system features (N = 60).

As shown in the survey results, expectations for future improvements were high. Specifically, 75% of respondents expected better medication management, 70% anticipated enhanced system efficiency and user interface, and 83.4% hoped for a reduction in prescription entry time. Additionally, 73.3% highlighted the importance of integrating a patient prescription history feature. These findings underscore the need for ongoing system refinement and stakeholder‐informed development (Figure 3).

### 3.6 Thematic Analysis of Open‐Ended Responses

Thematic analysis of the open‐ended responses revealed several recurring concerns and suggestions among participating physicians. A common theme was the absence of real‐time access to patients' prescription histories, which many participants indicated as a barrier to effective clinical decision‐making. Another frequently mentioned issue was the system's limited flexibility in accommodating diverse clinical workflows, particularly for physicians working in specialized fields. In addition, numerous respondents highlighted insufficient training, particularly in navigating advanced system features and handling technical problems, as a significant weakness. Frustration with login and data‐entry delays was also a dominant concern, with many physicians indicating that such technical inefficiencies disrupted their clinical efficiency. These qualitative insights provide valuable context to the quantitative results, shedding light on underlying challenges and informing potential areas for system improvement.

## 4 Discussion

Evaluation plays a pivotal role in system management, providing essential feedback on whether a system meets its operational and strategic objectives. This study was conducted in Mashhad and Sabzevar to assess physicians' satisfaction with the Social Security Organization's electronic prescription system. Since physicians are the primary end‐users of this system, their satisfaction and engagement are crucial to successful adoption and long‐term effectiveness.

The findings revealed that many participants believed the system had reduced prescribing errors and unnecessary interventions. This aligns with similar findings from a study in Singapore, where users agreed that electronic prescribing minimized medication‐related mistakes [16]. Studies in Saudi Arabia and Germany have also reported similar outcomes, reinforcing that digital prescribing can enhance safety and efficiency in clinical workflows [21, 28]. These results are consistent with broader evidence from Iranian settings [27], which highlights the potential of electronic prescribing to improve safety metrics while still facing operational challenges. Additionally, 73.4% of physicians expressed confidence in the system's confidentiality measures regarding patient data protection, a level of trust likely attributable to national‐level security infrastructure. One of the most appreciated features was the ability to transmit prescriptions directly from clinics to pharmacies, with 79.4% satisfaction. This aligns with the findings of Jebraeily et al., where this functionality scored the highest among system capabilities [27]. Similar features in Turkey and Saudi Arabia have been linked to significant workflow improvements [29]. Moreover, physicians with higher computer literacy were more optimistic about using the system, consistent with studies showing that digital fluency correlates positively with technology adoption [24].

This apparent contradiction—where 46.7% acknowledged the system's effectiveness in reducing prescription errors, yet the same proportion indicated they would not recommend it to colleagues—likely reflects dissatisfaction with other critical aspects of system use. Survey results show that 61.7% of physicians were dissatisfied with the time required to enter prescriptions and patient data, and 53.3% were dissatisfied with system login speed. Moreover, 55% expressed dissatisfaction with training related to handling system failures. Our results mirror experiences reported in other contexts, where the promise of reducing medication errors through electronic prescribing has been tempered by frustrations with system speed, user‐interface complexity, and inadequate support [27]. Such disconnects between perceived functionality and overall value have also been noted in other emerging healthcare contexts [25, 32]. This finding is consistent with those from other regions, where physicians cited lengthy delays in initiating electronic prescriptions as a barrier to their acceptance [33]. Other studies also confirm that interface speed and system response time are major predictors of user satisfaction [24].

These findings underscore that improving physician satisfaction with electronic prescribing systems involves more than just enhancing core functionalities. Issues such as insufficient training, low interface usability, and slow system performance point to gaps in both technical design and implementation strategy. To ensure wider acceptance and sustained usage, digital health initiatives must incorporate human‐centered design principles, responsive support infrastructure, and policy‐level commitments to continuous user education. This is particularly relevant in public sector settings where rapid, top‐down deployments may overlook end‐user needs. As such, physician feedback should play a central role in informing future updates and national digital health policies.

Regarding ease of use, only 36.7% of our respondents were satisfied, and 38.3% expressed dissatisfaction with the interface design. These results are consistent with findings by Fumis et al. [34], who reported user‐friendliness scores of 3.83 and 3.88 in their evaluation. A well‐designed user interface is essential for workflow efficiency and physician motivation to engage with the system [20]. Qualitative thematic analysis revealed that login authentication processes, slow data retrieval, lack of access to patients' prescription histories, and limited flexibility for specialized workflows were major contributors to dissatisfaction. Participants also cited frustration over insufficient training, emphasizing the need for faster login and prescription entry processes. The integration of qualitative thematic findings with quantitative survey results was intentional and designed to provide a richer, more comprehensive understanding of physician satisfaction. The qualitative data served as a complement to the numerical findings, helping to explain observed contradictions—such as high ratings for error reduction alongside reluctance to recommend the system—by revealing underlying usability and workflow concerns.

These concerns—especially the lack of real‐time access to patient medication history and limited adaptability of the system for different specialties—complement the quantitative findings and underscore key areas for improvement in usability, adaptability, and technical support. Understanding whether delays occur during system boot‐up, patient record access, or prescription entry is essential for guiding targeted technical improvements [16, 27, 34].

Given the suboptimal satisfaction with training and support services, targeted capacity‐building initiatives are essential. Studies have shown that ongoing technical support significantly improves physician engagement and system performance [17]. Based on our results, improving user interface design, integrating access to medical histories, and offering adaptive training modules would likely improve overall system performance and physician satisfaction.

However, only 36.7% of physicians believed the system improved patient care, and 43.4% thought it enhanced treatment quality. This suggests a potential disconnect between administrative functionalities (e.g., alerts, legibility) and clinical impact. Donyai et al. noted that system efficiency improvements do not necessarily translate to better care outcomes [19]. This issue has been observed in other developing healthcare systems, such as Vietnam, where data showed high functionality but limited influence on clinical outcomes [18]. Bridging this gap may require system redesign to integrate better clinical decision support and access to comprehensive patient medication histories [27].

### 4.1 Limitations

This study has several limitations. First, it was explicitly designed as an exploratory and context‐specific investigation in two northeastern cities (Mashhad and Sabzevar), with a small sample of 60 physicians. The focus was to capture initial usability patterns rather than to provide nationally generalizable findings, and therefore, statistical power is limited. Second, convenience sampling may have introduced selection bias, as more motivated respondents—either positively or negatively—might have been more likely to participate [27]. Third, the cross‐sectional design provides a snapshot of perceptions at a single point in time and does not capture how satisfaction may change over time, particularly with longer use or following system updates. Moreover, data were collected in 2021, and subsequent modifications to the electronic prescribing system may have influenced user experiences. Fourth, reliance on self‐reported measures introduces the possibility of recall errors and social desirability bias. Finally, although the questionnaire demonstrated strong internal consistency and underwent expert review for face validity, it has not been formally validated for content or construct validity against established acceptance models such as the Technology Acceptance Model (TAM). Future research should address these limitations by recruiting larger, multi‐regional samples using probabilistic sampling methods, incorporating objective system metrics (e.g., login times or error rates) alongside self‐reports, and conducting longitudinal studies to assess changes over time and further validate the measurement instrument. Overall, the present findings should be interpreted as preliminary, context‐specific insights that can guide subsequent large‐scale evaluations.

## 5 Conclusions

This cross‐sectional, exploratory survey found that physicians valued certain key features of the electronic prescribing system—particularly the ability to transmit prescriptions to pharmacies (79.4%) and maintain data confidentiality (73.4%)—but reported notable dissatisfaction with system speed, user interface design, and training quality. Although nearly half of the participants acknowledged the system's role in reducing prescription errors, an equal proportion (46.7%) indicated they would not recommend its use. This finding reveals a fundamental gap between the system's technical benefits and the actual user experience, underscoring that a system's full acceptance depends not just on its core functionality but also on its usability and performance.

This gap appears to be driven by major concerns over slow data entry and log‐in processes, limited interface usability, and insufficient training in managing technical problems.

Survey results provide opportunities to enhance the system and workflow, which is practical because these issues can affect the acceptability of new technology and the speed of dissemination in an organization. To directly address these root causes, we recommend prioritizing performance optimization to improve login and data entry speeds, undertaking a user‐centered redesign of the interface, integrating access to prescription history, and delivering modular, specialty‐specific training programs focused on both routine use and troubleshooting.

Since data collection occurred in 2021, future studies should account for possible system updates. Overall, these findings should be interpreted as preliminary, exploratory, and context‐specific insights based on a limited sample of 60 physicians in two cities. They highlight important usability barriers but are not intended for generalization to the national level. We recommend longitudinal, multi‐regional studies using objective system metrics (e.g., login duration, transmission success rate) to assess real‐world performance comprehensively. Such future research will help validate and expand upon the present findings, ultimately informing national‐level digital health strategies in Iran.

## Author Contributions

**Mohammad Reza Mazaheri Habibi:** design and critically revise the work for its important intellectual content. **Narges Norouzkhani:** interpretation of data, drafting the work. **Fatemeh Moghbeli:** analyse and interpret data, revising it critically for important intellectual content. **Mahdieh Mohammadi Majd:** acquisition, analysis, and interpretation of data, as well as drafting the work. **Gholamreza Moradi:** acquisition, analysis, and interpretation of data, as well as drafting the work. **Masood Setoodefar:** drafting and revising the work critically for important intellectual content. All authors have read and approved the final version of the manuscript. Mohammad Reza Mazaheri Habibi had full access to all of the data in this study and took complete responsibility for the integrity of the data and the accuracy of the data analysis.

## Disclosure

The lead author Masood Setoodefar affirms that this manuscript is an honest, accurate, and transparent account of the study being reported; that no important aspects of the study have been omitted; and that any discrepancies from the study as planned (and, if relevant, registered) have been explained.

## Ethics Statement

The Varastegan Institute for Medical Sciences Ethics Committee approved the protocols. The ethical approval number is IR.MUMS.fm. REC.1396.253. Informed consent was obtained from all subjects and/or their legal guardian(s). Relevant guidelines and regulations were followed in all experiments.

## Consent

The authors have nothing to report.

## Conflicts of Interest

The authors declare no conflicts of interest.

## Data Availability

Data and materials are available from the corresponding author upon reasonable request.
